# Stomatal and Photosynthetic Traits Are Associated with Investigating Sodium Chloride Tolerance of *Brassica napus* L. Cultivars

**DOI:** 10.3390/plants9010062

**Published:** 2020-01-02

**Authors:** Ibrahim A. A. Mohamed, Nesma Shalby, Chenyang Bai, Meng Qin, Ramadan A. Agami, Kuai Jie, Bo Wang, Guangsheng Zhou

**Affiliations:** 1 College of Plant Science and Technology, Huazhong Agricultural University, Wuhan 430070, China; iaa04@fayoum.edu.eg (I.A.A.M.); nas05@fayoum.edu.eg (N.S.); baicy@webmail.hzau.edu.cn (C.B.); qmm1713@webmail.hzau.edu.cn (M.Q.); kuaijie@mail.hzau.edu.cn (K.J.); zhougs@mail.hzau.edu.cn (G.Z.); 2 Faculty of Agriculture, Fayoum University, Fayoum 63514, Egypt; ramadanagami@yahoo.com

**Keywords:** *Brassica napus*, salinity, cluster analysis, stomata, growth, antioxidant enzymes

## Abstract

The negative effects of salt stress vary among different rapeseed cultivars. In this study, we investigated the sodium chloride tolerance among 10 rapeseed cultivars based on membership function values (MFV) and Euclidean cluster analyses by exposing seedlings to 0, 100, or 200 mM NaCl. The NaCl toxicity significantly reduced growth, biomass, endogenous K^+^ levels, relative water content and increased electrolyte leakage, soluble sugar levels, proline levels, and antioxidant enzyme activities. SPAD values were highly variable among rapeseed cultivars. We identified three divergent (tolerant, moderately tolerant, and sensitive) groups. We found that Hua6919 and Yunyoushuang2 were the most salt-tolerant cultivars and that Zhongshuang11 and Yangyou9 were the most salt-sensitive cultivars. The rapeseed cultivars were further subjected to photosynthetic gas exchange and anatomical trait analyses. Among the photosynthetic gas exchange and anatomical traits, the stomatal aperture was the most highly correlated with salinity tolerance in rapeseed cultivars and thus, is important for future studies that aim to improve salinity tolerance in rapeseed. Thus, we identified and characterized two salt-tolerant cultivars that will be useful for breeding programs that aim to develop salt-tolerant rapeseed.

## 1. Introduction

In agriculture, salinity is a great environmental problem causing stress that leads to a serious threat to plant growth and crop yield in many parts of the world, especially arid and semiarid regions; globally, 20% and 33% of cultivated and irrigated land, respectively, are salt-affected and degraded [1,2]. It is estimated that about half of the world’s land will be saline by the middle of the 21st century [1]. Morphology, physiology, and anatomy of plants are considerably affected by salt stress [3,4]. Salinity has multiple influences; besides malnutrition and accumulation of excess ions to potentially harmful levels in plants, it can cause drought stress [5,6]. Salinity causes disturbing impacts on all growth stages in plants beginning from a decline in seed germination as the first decisive and most sensitive stage, a slowing of plant development, decrease of shoot formation, decline of biomass production, decrease in crop yield, decrease the rate of survival and finally, plant death [6,7]. In addition, accumulation of NaCl in plants cells including stomatal guard cells, causing a reduction in guard cell size and rapid closure of stomata [6]. Previous studies have revealed that salt stress at the seedling stage reduced shoot and root dry weight, root and shoot length, and number of the leaves [8,9,10]. Seedling growth is very serious for the early foundation of plants under stress conditions, therefore, selecting cultivars that having rapid growth under saline conditions can contribute towards to ultimate growth and yield of plant species and cultivars. Consequently, this redoubled the importance of studying salinity tolerance in the seedling stage [11].

It is well established that salt stress has detrimental impacts on various key physiological and biochemical processes of plants, disrupting water relations, normal ion balance, and cellular homeostasis [3,4,6]. Besides osmotic stress and ion toxicity, salt-stress is also manifested as an oxidative stress at the subcellular level [12,13,14]. These three factors contribute to the negative effects produced by salinity in plants. The main processes such as proteins synthesis, photosynthesis, and lipid metabolis, are influenced during the beginning and increasing of salt stress within a plant. It promotes the overproduction of reactive oxygen species (ROS) like superoxide radicals (^•^O_2_^−^), hydrogen peroxide (H_2_O_2_), hydroxyl anions (^•^OH) and singlet oxygen (^1^O_2_) which trigger oxidative stress in plant tissues at subcellular level [13,14]. This oxidative stress causes decay of chlorophyll, reduces photosynthetic capacity, membrane lipid peroxidation, protein denaturation, and DNA damage [15,16]. Plants have adapted numerous advanced mechanisms to minimize oxidative injuries created by salt stress [6,7,17,18,19,20]. One of these mechanisms is protecting cellular and sub-cellular systems from cytotoxic effects of overproduction of ROS by enhancing activities both of enzymatic and non-enzymatic antioxidant defence systems in addition to accumulation compatible solutes [15,16,21,22,23,24]. It was reported that the level of antioxidant enzymes rises when plants are exposed to oxidative stress including salt stress [18,25,26]. Analysis of chlorophyll content and chlorophyll-based parameters are considered as an important way to evaluate the integrity during the photosynthetic process under salt stress, while the high performance of photosynthesis was reported to associate with salt tolerance ability [27,28]. In addition, biosynthesis of osmoprotectants and compatible solutes: e.g., proline and sugar) considered an adaptive role in mediating osmotic adjustment and protecting the sub-cellular structures in salt stressed plants [6]. 

Rapeseed (*Brassica napus* L.) is an annual plant belongs to the family of Brassicaceae. Globally, rapeseed production is the second-largest oil crop and world rapeseed production was estimated at a record 71.5 million tonnes in the 2016/2017 season [29]. Although rapeseeds classified as semi-tolerant plants [8], water and soil salinity are the greatest extensive problem for rapeseeds production [19]. Breeding and deployment of tolerated genotypes that have good performance under salt stress, particularly in the seedling stage can be an appropriate solution to maintain acceptable yield in the face of salt stress [17]. Therefore, it is of a great significance to develop salt-tolerant rapeseed cultivars that are needed to provide a partial solution to increase salt accumulation problem. Studying of morpho-physiological, biochemical, and anatomical response to salt stress could be helpful to further understand the molecular tolerance traits [30] and will provide fundamental knowledge to develop salt-tolerant genotypes. It is well reported that tolerance to salinity can greatly differ among varieties or cultivars of single plant species; therefore, selection of high salinity tolerance cultivars is crucial to further improve salinity tolerance. The genetic variability present in rapeseed could be exploited to evaluate and screen for high-salinity tolerance. Various screening methods of salinity tolerance have been developed in rapeseed based on multi-index results [10,23,31,32]. The membership function value (MFV) is an appreciate screening method for different stress conditions; drought stress [33,34], heat stress [35], nitrogen stress [36], and salt stress [31]. Wu et al. [31] evaluated the salt tolerance of *B. napus* germplasm using the MFV method in the germination stage but an effective screening method in the seedling stage not presented. Thus we used MFV as a new method for screening salinity tolerance of rapeseed cultivars in the seedling stage, in addition, determine the role of photosynthetic and stomata anatomical traits in salinity tolerance of rapeseed plants. The major aims are: (1) to understand the effect of NaCl on growth and biomass yield of rapeseed cultivars, (2) to determine variations in the degree of salinity tolerance among rapeseed cultivars, (3) to screen out the salt-tolerant and salt-sensitive cultivars for future studies, (4) in addition, to determine the relationship between photosynthetic and stomata anatomical traits with salinity tolerance of rapeseed cultivars.

## 2. Results

### 2.1. Seedling Growth and Biomass Yield

**Figure 1:**
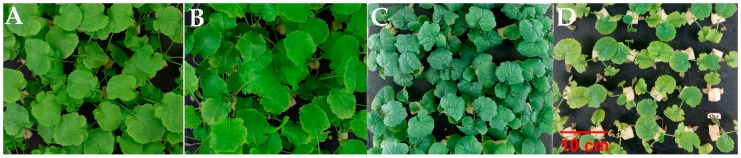
The symptoms of seedlings after 28 days grown under control (**A**,**B**; without NaCl) and 200 mM NaCl treatment (**C**,**D**); A and C: Hua6919 cultivar; B and D: Zhongshuang11 cultivar. bars = 10 cm.

Significant reduction (*p* < 0.05) was noticed in growth in terms of plant height (PH), number of leaves plant^−1^ (NL), total leaves area plant^−1^ (TLA), shoot fresh weight (SFW), root fresh weight (RFW), shoot dry weight (SDW), and root dry weight (RDW) of the rapeseed cultivars subjected to 100 or 200 mM of NaCl stress (Figure 1, Tables S1 and S2). 

Without the adding of NaCl, maximum and minimum values of PH (60.1 and 48.9 cm) were observed in Ningza27 and Fengyou520, respectively. With adding 100 or 200 mM NaCl, there was a significant decline in plant height in all cultivars, however, the intensity varied among varieties. This decline was more severe under 200 mM than 100 mM treatment. The decrease in PH varied from minimum of 5% in Fengyou520 to as high as 22% in Yangyou9 under 100 mM of NaCl treatment. The drastic reduction in PH to the extent of 39 and 38% occurred in Yangyou9 and Ningza27, respectively, while, the minimum decrease was observed in Fengyou520 (18%) and Hua6919 (23%) under 200 mM of NaCl (Table S1). Salt tolerance coefficient (STC) varied among rapeseed cultivars based on PH and ranged from 69.2% in Yangyou9 to 88.5% in Fengyou520 (Table 1).

Total leaves area (TLA) and number of leaves (NL) of rapeseed seedlings were also significantly (*p* ≤ 0.05) decreased in all the cultivars as compared to control when exposed to NaCl treatments of 100 and 200 mM. The results presented in Supplementary Table S1 indicate that the decline was more at higher doses of NaCl application (200 mM). This decline was higher in TLA than the decline in the NL under the applied NaCl treatments. Comparing the mean reduction in TLA under two NaCl treatments, all rapeseed cultivars responded differently, however the highest reduction was found in Zhongshuang11 (61%) followed by Ningza27 (56%) and the lowest reduction was noted in Fengyou520 (39%) followed by Hua6919 (43%) as compared to control, while the highest reduction in LN was found in Yangza11 and Yangyou9 (30%) and the lowest reduction was noted in Yunyoushuang2 (11%) followed by Hua6919 (12%) as compared to control. Data in Table 1 revealed that the mean of salt tolerance coefficient (STC) varied among rapeseed cultivars based on TLA and NL. STC based on TLA ranged from 39.1% (Zhongshuang11) to 60.6% (Fengyou520), while based on NL, it ranged from 70.3% (Yangza11) to 89.1% (Yunyoushuang2). 

The adding of NaCl also reduced shoot fresh weight (SFW) and root fresh weight (RFW) of seedlings in all rapeseed cultivars, as shown in Supplementary Table S2 to the extent of 57% in Ningza27 and 48% in Xingyouza553 under 100 mM, while under 200 mM of NaCl, the maximum reduction was noted in Ningza27 (68%) and Zhongshuang11 (59%), respectively. The minimum reduction in SFW and RFW was noted in Yunyoushuang2 (35%) and Hua6919 (21%) under 100 mM NaCl, while under 200 mM NaCl it was in Hua6919 (54 and 45%), respectively. NaCl induces a great decrease in shoot dry weight (SDW), and root dry weight (RDW) of seedlings in all rapeseed cultivars, under 100 mM of NaCl the reduction in shoot and root dry weight varied from 17 and 21% in Hua6919 to 47 and 47% in Zhongshuang11, respectively. At 200 mM NaCl, reduction in shoot and root dry weight varied from 34% and 48% in Hua6919 to 62 in Zhongshuang11 and 64% in Yangyou9, respectively (Table S2). Means of salt tolerance coefficient of seedling SDW and RDW appeared to vary depending upon cultivars and maintained that Hua6919 cultivar has the highest values (Table 1).

### 2.2. Chlorophyll Content (SPAD)

An increase in total chlorophyll content (SPAD) was observed in some rapeseed cultivars at the seedling stage (Figure S1), while there a decrease was noted in other cultivars. Fengyou520 exhibited a considerable increase in SPAD (134.4%) closely followed by Hua6919 (127.4%) whereas a noticeable lowering in SPAD (21.3%) was recorded for Yangyou9 and Xiangyouza553. The highest reduction was noted in Yangyou9 cultivar by 9 and 22% under 100 and 200 mM NaCl, respectively, as compared to the control. The salt tolerance coefficient of SPAD values ranged from 85 to 129% with Fengyou520 achieving the highest in that index (Table 2). 

### 2.3. K^+^, Total Soluble Sugar (TSS) and Proline Concentrations (ProC)

A great reduction was observed in K^+^ of rapeseed cultivars under salt stress conditions (Figure S2A). However, K^+^ decreased with increasing of salinity. The highest decrease values were achieved by Zhongshuang11 (81%) and Xiangyouza553 (88%), while the lowest decrease values were achieved by Fengyou520 (70%) and Hua6919 (77%) under 100 and 200 mM of salt stress, respectively. STC mean based on K^+^, Hua6919 ranked as the highest one, while Xiangyouza553 as the lowest one (Table 2). TSS and ProC increased in the rapeseed leaves as compared to plants with no salt stress and it showed significantly (p ≤ 0.05) different among the rapeseed cultivars (Figure S2B,C). At 100 and 200 mM NaCl treatments, the range of TSS increasing was from 6 and 31% in Xiangyouza553 and Ningza27 to 47 and 78% in Fengyou520 and Hua6919, respectively. Hua6919 and Huashuang5 under 200 mM NaCl showed the highest value in TSS (146 and 144 mg g^−1^ DW) compared to those of the other cultivars. At 100 and 200 mM NaCl treatment, the range of ProC in leaves was from 24% and 55% in Hua6919 and Zhongshuang11 to 115% and 130% in Rongyou18 and Hua6919, respectively. Remarkably, Zhongshuang11 and Yangyou9 exhibited a slight increase in proline accumulation (1.46 and 1.58 folds). The overall cultivars salinity tolerance coefficient based on TSS indicated that Fengyou520 and Hua6919 have the highest values, while Ningza27 had the lowest values. Based on ProC, Fengyou520, and Yunyoushuang2 had the highest values, while Zhongshuang11 and Yangyou9 had the lowest values (Table 2).

### 2.4. Relative Water Content (RWC) and Electrolyte Leakage (EL)

A great reduction was observed in RWC of rapeseed plants under salt stress condition, however RWC decreased with increasing of salinity (Figure S3A). At the highest salt level, the cultivars Hua6919 was successful in maintaining the lowest decrease RWC (14%) and the highest decrease values were achieved by Zhongshuang11 (31%) and closely followed by Yangyou9 (28%). The salt stress markedly increased EL% in all rapeseed cultivars which generated a great variation in EL%. An increase in NaCl concentrations to 100 or 200 mM of NaCl significantly increased EL% over the control (Figure S3B). Under the highest salt stress level, EL in Hua6919 and Yunyoushuang2 was increased (127% and 149%, respectively) whereas highly increased was noted in Zhongshuang11 and Huashuang5 (208% and 182%, respectively) as compared to the control. Results showed that higher EL was recorded in the salt susceptible cultivars. It is clear that the increasing of EL is a symptom of salt stress injury and indicator for salt sensitivity. The overall cultivars, the STC based on RWC, Hua6919 ranked as the highest one, while Zhongshuang11 as the lowest one. In contrast with the STC based on EL%, Zhongshuang11 ranked as the highest one, while Hua6919 ranked as the lowest one (Table 2).

### 2.5. Enzymatic Antioxidants

The results showed a positive relationship between NaCl levels and antioxidant enzyme activities in rapeseed cultivars, and there were significant (*p* ≤ 0.05) differences among cultivars in response to NaCl treatments. The activity of SOD in all the cultivars was significantly (*p* ≤ 0.05) increased when exposed to NaCl concentrations as compared to control. Under 100 mM of NaCl, the maximum activity of SOD was observed in Hua6919 (134%), while the minimum increase was recorded in Zhongshuang11 (38%). At 200 mM treatment, increasing SOD activity varied from 43% in Rongyou18 to 156% in Hua6919 than control, respectively (Figure S4A). The maximum CAT activity was recorded in Rongyou18 (83%) and Hua6919 (82%) under 100 mM NaCl as compared to control, while under 200 mM NaCl, it was Fengyou520 (115%) and Rongyou18 (83%) higher as compared to control. On the other hand, cultivars Zhongshuang11, Huashuang5, and Yangyou9 had a lower increase in CAT activity (24, 30, and 37%, respectively) under 100 mM NaCl, while under 200 mM NaCl, it was Yangyou9, Ningza27, and Huashuang5 (45, 46, and 47%, respectively) (Figure S4B). Ascorbate peroxidase (APX) activity increased significantly (*p* ≤ 0.05) in rapeseed seedlings with the increase in salt stress levels in all cultivars except in Yangyou9, Yangza11, and Fengyou520 under 100 mM of NaCl and Yangyou9 under 200 mM NaCl (Figure S4C). The maximum APX increase was recorded in Hua6919 (36%), while Yangyou9 showed a decrease in APX activity as compared to control under 200 mM. The minimum POD activity increase was recorded in Zhongshuang11 (10%) and Ningza27 (27%), while the maximum increase was recorded in Huashuang5 and Yunyoushuang2 (75 and 71, respectively) compared to control under 100 mM of NaCl (Figure S4D). The increase in POD activity was measured about 123% in Fengyou520 under 200 mM of NaCl, while Xiangyouza553 and Yangza11 showed minimum increase under 200 mM NaCl. Data in Table 2 revealed that the mean of salt tolerance coefficient (STC) varied among rapeseed cultivars based on SOD, CAT, APX, and POD activities. STC based on SOD ranged from 130% (Huashuang5) to 245% (Hua6919), while based on APX, it ranged from 94% (both of Yangyou9 and Yangza11) to 121% (Hua6919). STC based on CAT and POD ranged from 137% and 128% (Xiangyouza553) to 195% and 193% (Fengyou520), respectively.

### 2.6. Ranking and Grouping of Rapeseed Cultivars for Salt Tolerance Evaluation

In order to assess the patterns of variation in salinity tolerance among rapeseed cultivars, all morphological, biochemical, and physiological traits were standardized for salinity tolerance evaluation based on salinity tolerance coefficients (STC) or indexes. STC of all traits is presented in Table 1 and Table 2. STC was further used to calculate the membership function values (MFV) of rapeseed cultivars. Furthermore, Pearson’s correlation analysis was performed to determine the relationship (if any) among STC based on the different traits. STC relating to several parameters show significant correlations. The results revealed that plant height (PH) was strongly positively correlated with SDW, TLA, POD, SPAD, and TSS (*p* < 0.01) and SFW, RFW, CAT, K^+^, ProC, and RWC (*p* < 0.05) and it was negatively correlated with EL. SDW displayed positive correlations to the PH, SFW, RFW, RDW, TLA, SPAD, K^+^, POD, TSS, ProC, and RWC (*p* < 0.01) and NL, SOD, CAT, and ProC (*p* < 0.05), but showed a negative correlation with EL. SPAD was also positive highly correlated with PH, SDW, RDW, POD, K^+^, ProC, and RWC (*p* < 0.01) and RFW, TLA, CAT, and TSS (*p* < 0.05), while it was negatively correlated with EL. On another way, EL had negative correlations with the all traits such as PH, SFW, RFW, SDW, RDW, TLA, APX, CAT, POD, SPAD, ProC, RWC, and TSS but significantly negative correlations with SOD (*p* < 0.01) and NL (*p* < 0.05). Hence, all growth and biomass and physiological and biochemical traits were used to evaluate the NaCl tolerance of all rapeseed cultivars.

**Figure 2:**
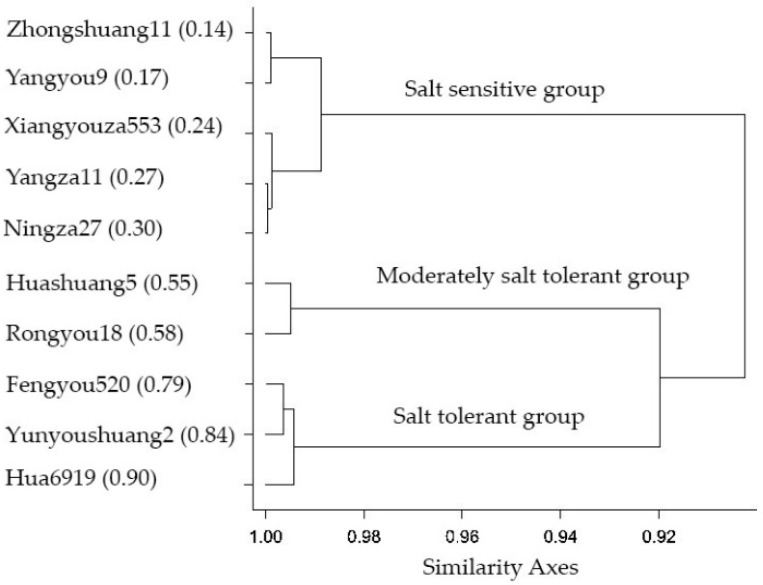
Dendrogram from cluster analysis for salinity tolerance showing the classification of 10 *B. napus* L. cultivars based on the membership function value (MFV) of morpho-physiological and biochemical indices into three groups. Values surrounded by parenthesis is the mean of MFV of each cultivar listed after the cultivar name.

For the aim of ranking the degree of salinity tolerance among rapeseed cultivars, STC was used to calculate the MFV. The higher the mean of MFV, the higher salt tolerance. Hua6919 and Yunyoushuang2 cultivars had higher mean of MFV (0.90 and 0.84), respectively. By contrast, Yangyou9 (0.17) and Zhongshuang11 (0.14) showed lower mean of MFV (Table S3). Based on this result, Hua6919 and Yunyoushuang2 were ranked 1 and 2, whereas Yangyou9 (0.18) and Zhongshuang11 were ranked 9 and 10, respectively (Table S3). Furthermore, Euclidean distance cluster analysis was used for grouping of rapeseed cultivars based on the MFV calculations. All 10 rapeseed cultivars were grouped into three distinct clusters at 0. 92 similarity coefficient level (Figure 2). Cluster I (salt-tolerant group) was formed with Hua6919, Yunyoushuang2 and Fengyou520. Rongyou18 and Huashuang5 were in Cluster II (moderately salt-tolerant). Cluster III denoted the salt-sensitive group, which was composed of Zhongshuang11, Yangyou9, Xiangyouza553, Ningza27, and Yangza11. Our results suggested the highly positive correlation between STC based on SPAD values with most of the growth, biomass, physiological, and biochemical traits, indicated its importance in salinity tolerance of rapeseed cultivars. The 10 cultivars were further evaluated by photosynthetic gas exchange and stomata anatomical traits to assess its role in salinity tolerance of rapeseed cultivars.

### 2.7. Assessment Role of Photosynthetic Gas Exchange Parameters and Stomata Anatomical Traits for Salinity Tolerance of Rapeseed Cultivars 

Net photosynthesis rate (Pn), transpiration rate (Tr), stomatal conductance (Gs), and stomata anatomical traits were further evaluated for all cultivars to assess its role in rapeseed salinity tolerance. Pn, Tr, and Gs in salt-stressed plants greatly varied among the 10 rapeseed cultivars (Figure S5). A decline in Pn, Tr, and Gs was observed for all rapeseed cultivars. The 200 mM was more harmful compared to the 100 mM of NaCl. Under high salinity level, analysis of results showed that the highest reduction in Pn, Tr, and Gs was achieved in Zhongshuang11 (67, 69, and 65%, respectively) whereas the lowest minimizing (35, 31, and 36%, respectively) was found in Hua6919 (Figure S5). However, the salt tolerance coefficient of all cultivars referred that, Hua6919 had the highest values based on Pn, Tr, and Gs (75.6, 74.7, and 67.3%, respectively), while Zhongshuang11, Xiangyouza553, and Zhongshuang11 (43.6, 42.9, and 32.2%, respectively) had the minimum values (Table 3). 

**Figure 3:**
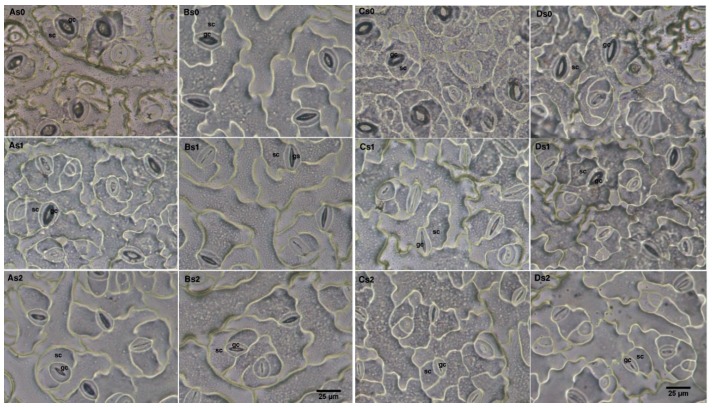
Photographs of *B. napus* L. leaf abaxial surface grown under different salt stress levels. (**A**) Hua6919; (**B**) Yunyoushuang2; (**C**) Yangyou9; (**D**) Zhongshuang11 cultivar; S0 (0 mM NaCl); S1 (100 mM NaCl); S2 (200 mM NaCl); gc, guard cells; sc, subsidiary cells, bar = 25 μm.

Light microscopy observation of the lower surface (abaxial side) of rapeseed cultivars leaves created by different salinity levels revealed significant changes in anatomical structures (Figure S6; Figure 3). Considerable variation in stomata aperture area (SAA) and stomatal density (SD) of 10 rapeseed cultivars leaves was noticed among the treatments of salt stress. Salt-stressed plants showed a great reduction in SAA due to the application of 100 and 200 mM NaCl, in comparison with the control. Salt stress recorded a considerable decrease in SAA with the increase in salinity levels the highest decrease observed in the most sensitive cultivars; Zhongshuang11 (63 and 85%) and yangyou9 (46 and 81%) under 100 and 200 mM NaCl treatments, respectively, while the minimum decrease was observed in the most tolerant cultivars Hua6919 (12 and 33%) and Yunyoushuang2 (22 and 48%), respectively, as compared to control (Figure S6A; Figure 3). Salt stress recorded a considerable increase in SD with the increase in salinity level. By contrast, results showed that in general, the cultivars having a higher reduction in leaf area recorded a larger increase in stomata density. Results also showed that higher SD was noted in the salt susceptible cultivars. It was clear from the mean results in Supplementary Figure S6B that SD was a symptom of salt stress injury and indicator for salt-sensitive, so it was considered as salt sensitive index. Hua6919 cultivar revealed greatest salinity tolerance coefficient (77.4%), while Zhongshuang11 cultivar showed the lowest STC based on SAA (26.0%) (Table 3).

**Figure 4:**
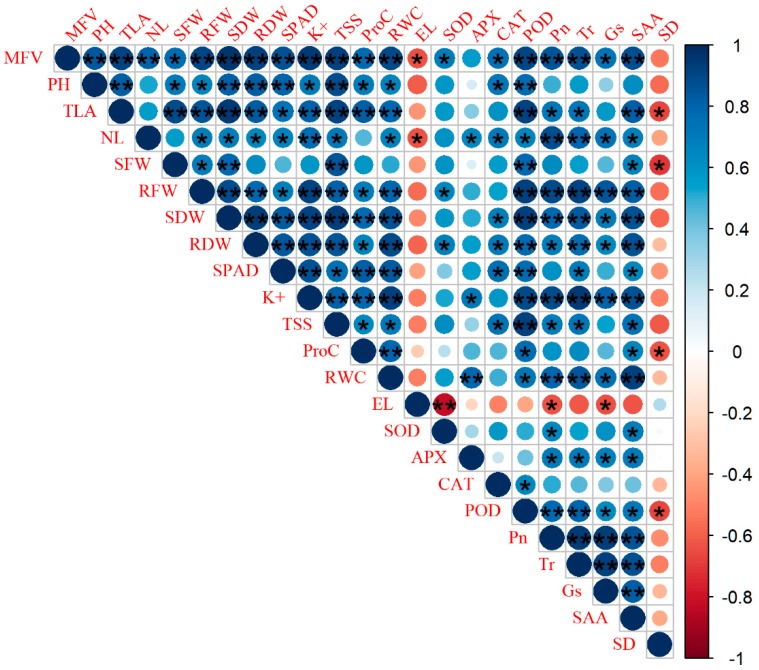
Pearson’s correlation among salt tolerance coefficients based on the different parameters from 10 *B. napes* cultivars exposed to different salt stress levels (0, 100, and 200 mM NaCl). Each circle indicates the Pearson’s correlation coefficient of a pair of parameters. MFV; mean of MFV, PH; plant height, TLA; total leaves area, NL; number of leaves, SFW; shoot fresh weight, RFW; root fresh weight, SDW; shoot dry weight, RDW; root dry weight, K^+;^ potassium concentration, ProC; proline concentration, TSS; total soluble sugar, RWC; relative water content. EL; electrolyte leakage, SOD; superoxide dismutase, APX; ascorbate peroxidase, CAT; catalase, POD; peroxidase, (Pn; net photosynthetic rate, Tr; transpiration rate, and Gs; stomatal conductance, SAA; stomata aperture area, and SD; stomatal density). * and **; Correlation is significant at the 0.05 and 0.01 levels, respectively.

STC relating to the photosynthetic gas exchange and stomata anatomical traits showed highly significant correlations with the mean of MFV and STC based on several morpho-physiological and biochemical traits. The mean of MFV was positive correlated with STC of Pn, Tr, and SAA (*p* < 0.01) and Gs (*p* < 0.05) Figure 4. This result indicated great impotence of SAA, Pn, and Tr in rapeseed salinity tolerance. Among the photosynthetic gas exchange and anatomical traits, the SAA was the most highly correlated with mean of MFV, morpho-physiological, and biochemical indices. Overall, our results suggested that SAA highly correlated with salinity tolerance in rapeseed cultivars and could be a reliable trait indicated its importance for improving rapeseed salinity tolerance. 

## 3. Discussion

Salinity in soil and water is a global problem that severely threatens food production. In this study, we used MFV based on different morpho-physiological and biochemical traits as a new method for screening salinity tolerance of rapeseed cultivars. In addition, we used photosynthetic traits and the anatomical traits of stomata in rapeseed plants to study the increase in salt stress at the seedling stage. Salt stress decreased the growth performance of seedlings from all cultivars. A plant’s ability to resist salt stress varies widely among different species and cultivars [17]. Indeed, we found that salt stress influenced various morpho-physiological, anatomical and biochemical traits; such as growth stunting, alterations in stomatal density, a decline in tissue water content, and chlorophyll content. These effects coincided with the overproduction of ROS, such as ^•^O_2_^−^, H_2_O_2_, ^•^OH, and ^1^O_2_ and thus, led to increases in oxidative stress [37,38]. Plants adopt many strategies to tolerate salt stress, such as altering their ion homeostasis, osmotic potential, and enhancement of the antioxidant defense system. This knowledge can be used by breeding programs to develop more salt tolerant genotypes [4,37,39,40].

Our study showed that the growth traits of rapeseed cultivars and consequently rapeseed shoot and root biomass markedly decreased as salt levels increased (Tables S1 and S2; Figure S1). Striking differences were observed among the rapeseed cultivars Zhongshuang11 and Yangyou9, which were the cultivars that were the most severely affected by salt stress. However, Hua6919 and Fengyou520 were resistant to the NaCl treatments. These findings are consistent with Tatar et al. [41] who reported that salt stress significantly reduced the total dry matter of rice cultivars. Similarly, Chunthaburee et al. [42] reported that salt stress significantly reduced both the fresh and dry weight of the salt-sensitive rice cultivar IR29 at the seedling stage. Salt stress is expected to negatively affect water absorption by seedling roots leading to osmotic stress, ion toxicity, and/or an ionic/nutritional imbalance [24]. These salt-induced effects inhibit the development of the root and shoot of rapeseed plants and thus, negatively affect rapeseed shoot and root yields. The maximum dry mass in Hua6919, Fengyou520, and Yunyoushuang2 may be explained by a more efficient detoxification mechanism, including antioxidant defense mechanisms. 

When plants are grown in unfavorable conditions, chlorophyll levels are a good indicator of photosynthetic activity [43]. In our study, aggravated salt stress caused significant changes in chlorophyll content (SPAD) in most rapeseed cultivars. In some cultivars, the SPAD value decreased as salt stress increased. Indeed, in salt sensitive plants, salt stress typically induces leaf burning and the degradation of pigments [44]. We found that cultivars capable of increasing chlorophyll content when experiencing salt stress were tolerant cultivars. This result is consistent with the previous finding that chlorophyll content is a biochemical marker for salt tolerance in plants [45,46,47]. Due to the high variation among rapeseed cultivars in chlorophyll content, we further studied the influence of photosynthetic gas exchange and anatomical traits in rapeseed salinity tolerance.

In contrast to Na^+^, K^+^ is the most abundant cation in plant cells and an essential nutrient that is important for both ionic and pH homeostasis. K^+^ is also important for maintaining adequate membrane potential and many biochemical processes [48]. The uptake and distribution of K^+^ and Na^+^ are key determinants of plant salinity tolerance. Our results indicate that salt-tolerant cultivars (Hua6919 and Yunyoushuang2) retain more K^+^ in their leaves than the salt-sensitive cultivars (Zhongshuang11 and Yanyou9) when they experience salt stress Table 2. The findings in our study are in good agreement with previous studies [49,50]. More tolerant cultivars that contain intrinsically higher K^+^ levels in their leaves may be indicative of several possible mechanisms. For example, a higher capacity for K^+^ uptake promotes photosynthesis under salt stress, activates enzymes, and reduces the amount of ROS in photosynthetically active mesophyll tissue. A higher capacity for K^+^ uptake also adjusts stomatal function, protein synthesis, cell osmoregulation, oxidant metabolism, and turgor maintenance. Apart from ion homeostasis, proline, and sugar accumulation is another well-known mechanism that has evolved in many plant species to overcome salt stress. The accumulation of sugars and proline in salt stress conditions protects the cells by balancing the osmotic strength of the cytosol with that of the vacuole and the external environment [51,52]. Furthermore, proline has a double role in enhancing salt-stress tolerance. Similar to peroxidases, proline can scavenge reactive oxygen species [20,51]. Consequently, TSS and proline accumulation is one of the most important physiological indices for salt stress tolerance in plants [53]. Our results show that salt stress greatly increased the accumulation of TSS and proline in the leaves of ten rapeseed cultivars (Figure S2). We found that the accumulation of TSS and proline in rapeseed cultivars subjected to salt stress correlated with salt tolerance (Figure S2; Table 2). Although proline content significantly increased in the leaves of the salt-tolerant and salt-sensitive cultivars experiencing salt stress, more proline accumulated in the tolerant cultivars than in the sensitive cultivars [54,55] and in rice cultivars [21]. The accumulation of organic solutes—mainly sugars—are the major solutes involved in osmotic adjustment in glycophytic plants exposed to osmotic and salt stress [56]. The increase in the levels of soluble sugars and proline in the tolerant cultivars probably has a role in osmotic adjustments, improving water uptake and maintaining RWC in plants experiencing stress. These changes promote the stability of membranes, prevent membrane fusion, and maintain protein activity. These mechanisms helped the plant to avoid tissue death and thereby enable the continued development and growth in saline conditions. 

RWC is a simple parameter that can be used for screening plant salinity tolerance and a key marker for salt stress studies. When RWC can be preserved in cells and tissues, it allows a resumption of metabolic activity by osmotic adjustments and other physiological traits associated with salinity tolerance [57]. We found that healthy and tolerant rapeseed cultivars with low salt-induced injuries maintained a high RWC. The reduction in the growth of rapeseed cultivars grown in salt-stress conditions was associated with a reduction in the RWC and an increase in EL (Figure S3). Plasma membranes are the primary site of ion-specific salt injury. Therefore, EL from the plasma membranes is an important screening tool for identifying salt-tolerant cultivars [58]. Under salt stress, an increase in EL was reported and attributed to the loss of cell turgor due to limited water availability for cell expansion [58]. Accumulation of ROS is usually accompanied by the stress-induced EL and often a consequence of programmed cell death [58]. In the present study, increases in EL were observed to be higher in the salt-sensitive cultivars than in the salt-tolerant cultivars. These findings are in good agreement with many researchers who found that salt stress enhanced EL in the salt-sensitive cultivars of rapeseed [59], japonica rice [60], or other salt-sensitive species [61]. 

In this study, low increases in EL were found in the salt-tolerant cultivars with the activation of SOD, POD, CAT, and APX under salt stress (Figure S4). Increases in the levels of plant antioxidant defenses were shown to be positively correlated with reduced oxidative damage and improved salinity tolerance [22,26,62,63]. Rapeseed cultivars with either inducible or constitutively high antioxidant levels have better resistance to this oxidative damage. Higher values of STC based on SOD, CAT, POD, and APX activities in salt-stressed Hua6919 show that this cultivar has a higher ROS scavenging capacity relative to other cultivars when it experiences salt stress, which correlated with lower EL values than in the salt-sensitive cultivars (Table 2).

The salt tolerance of rapeseed was evaluated comprehensively by cluster analysis using MFV values. The results showed that rapeseed cultivars had different sensitives to salt stress. Cultivars with higher MFV (Hua6919 and Yunyoushuang2; 0.90 and 0.84, respectively) were classified as salt-tolerant. In contrast, cultivars with the lower MFV values (Yangyou9 and Zhongshuang11; 0.17 and 0.14, respectively) were classified as salt-sensitive. Tolerance at the adult stage is correlated with tolerance at the seedling stage. The main objective of rapeseed breeders is to develop salt-tolerant varieties. The results from our study indicate that stress tolerance indices explain some of the tolerance to salt stress. The data on the morpho-physiological and biochemical indices can be used to screen rapeseed germplasm for salt tolerance.

Due to the high variation among rapeseed cultivars in chlorophyll content, we further studied the role of photosynthetic gas exchange and the anatomy of stomata in rapeseed salinity tolerance. Our results showed negative effects of salinity on the net photosynthetic rate (Pn), transpiration rate (Tr), and stomatal conductance (Gs) of rapeseed cultivars (Figure S5; Table 3). This finding was compatible with Akhtar et al. [64] and Ma et al. [44] who reported that the net photosynthetic rates and transpiration rates were reduced significantly when salinity was increased. It is clear from the results, that the photosynthetic capacity is lowered, due to osmotic stress and partial closure of the stomata. Transpiration performs an important physiological role in maintaining the osmotic concentration in the plant. Severe salt stress caused a greater reduction in photosynthetic gas exchange parameters in the salt-sensitive relative to the salt tolerant cultivars. These findings are consistent with Nounjan et al. [27] who reported that protecting the photosynthetic process from damage led to high-performance photosynthesis and thus salt tolerance in rice CSSL lines. Our result showed that strong correlation between SAA and the Pn, Tr, and Gs. The tolerant cultivars decrease the reduction in Pn, Tr, and the CO_2_ assimilation rate by increasing SAA than the sensitive cultivars. Thus, salinity may affect photosynthesis by influencing the stomata. Based on our results, a significant correlation between the mean of MFV and the Pn, Tr, and Gs indicate its importance in rapeseed salinity tolerance (Figure 4).

Stomata are highly sensitive to environmental stress conditions and may change their number and distributions. Salt-stressed plants showed a great reduction in SAA but SD was increased by increases in salinity (Figure S6; Figure 3). Our results show that a strong correlation between SAA and salinity tolerance, based on physiological and biomass yield traits and that SAA is responsible for the superior performance of salt tolerant rapeseed cultivars (Figure 4). Our results also indicate that the size of guard cells was highly variable and changes in response to salt stress. In line with these results, Larcher et al. [65] reported that leaves that developed during drought usually have smaller stomata than leaves that develop under well-watered conditions. Also, Waqas et al. [66] reported that SAA declined on the adaxial and abaxial surfaces of quinoa leaves in response to salt stress. In the present study, the relative changes (% of control) of the SD of salt-tolerant cultivars negatively correlated with the mean of MFV. SAA was observed to be higher in the salt-tolerant than in the salt-sensitive cultivars. This could be explained by salt stress causing a decrease in the size of epidermal and subsidiary cells for sensitive rapeseed cultivars during stomatal development [67,68]. Indeed, Hu & Schmidhalter [69] found that salt stress causes a reduction in the widths and lengths of epidermal cells. Salt stress causes a greater decrease in RWC, turgor pressure and cell volume of the guard cells in the salt-sensitive cultivars. This difference led to a reduction in Pn, Tr, and the CO_2_ assimilation rate. Our results showed that SAA was significantly correlated with Pn and Tr and that the decreases in Pn, Gs, and Tr that we observed during salt stress were largely dependent on stomatal closure (Figure 4). Since stomatal closure has negative effects on CO_2_ uptake, photosynthesis, transpirational cooling, as well as water and nutrient uptake, the enhancement of these effects in the salt-sensitive cultivars contributes to salt sensitivity. Cultivar-specific anatomical traits, such as larger stomata aperture area (open stomata) contribute to a high water potential in their tissues that contributes to metabolic activity through osmotic adjustments during growth in stressful conditions and thus, contribute to higher salt tolerance [70]. In the tolerant cultivars, greater SAA promotes higher levels of Pn, Tr, and CO_2_ assimilation rates than in the sensitive cultivars. In our study, we found that stomata aperture area (SAA) is a reliable indicator of salt stress tolerance in rapeseed and could be used as indicator of salt tolerance. This study provides evidence that photosynthetic gas exchange and stomata anatomical traits have an important role in the salinity tolerance of rapeseed cultivars.

## 4. Materials and Methods 

### 4.1. Materials, Conditions of Growth, and Experimental Treatments

The experiments were conducted under controlled conditions in a greenhouse located at Huazhong Agricultural University, China between March 2018 and August 2018. The treatments were a factorial arrangement of 10 cultivars and 3 NaCl levels. The experiment design was a randomized block and each treatment was replicated three times. The rapeseed (*Brassica napus* L.) cultivars were Zhongshuang11, Yangyou9, Hua6919, Xiangyouza553, Yangza11, Fengyou520, Huashuang5, Ningza27, Rongyou18, and Yunyoushuang2. These cultivars were grown hydroponically under three NaCl levels, i.e., 0 (S0) as a control, 100 (S1), and 200 (S2) mM NaCl. Uniform seeds of *B. napus* cultivars were surface sterilized with 70% ethanol for 5 minutes and followed by washing with distilled water five times. The seeds were placed on cotton gauze that floated over the deionized water for the germination. After 7 days from germination, 42 uniform seedlings from each cultivar were transplanted into foam board (polystyrene, 60 × 40 cm, length × width) suspended in the black plastic basin (60 × 40 × 10 cm, length × width × height) containing 10 L of modified Hoagland’s nutrient solution [71]. To avert seedlings osmotic shock, NaCl was dissolved in the nutrient solution and it was increased to the desired levels by incremental additions of NaCl over a 6-day period. The pH of the solution was adjusted to be 6.5 using diluted KOH or HCl before refreshing. Each seedling was anchored to pore (2 cm diameter (in the foam board with a sterilized sponge. Each foam board had 6 rows of pores, and each row had 7 pores for a total of 42 seedlings per board. The distance between two seedlings was 3.6 cm (between rows) and 4.0 cm (within a row), respectively. To support the seedling, pieces of square sponge about 1.5 cm and cut the sponge square along a diagonal line. The temperature, light/dark cycle and relative humidity of the greenhouse were 23 ± 2 °C, 16/8 h, and 75 ± 5%, respectively. The nutrient solution in the black plastic basin was continuously aerated using air pumps until the end and replaced every 7 days and each tank was moved randomly to a new location. The experiment was repeated 3 times under the same conditions. All chemicals used were of analytical grade, procured from Sinopharm Chemical Reagent Co., Ltd. (Shanghai, China).

### 4.2. Assessment of Growth and Biomass Yield

Measuring different traits were conducted 28 days after imposing salt stress (35 days after germination). Six seedlings were used for measuring morphological parameters from each replicate and plant height was measured. The number of leaves per plant was counted manually and total leaves area was recorded by an LI-3000 portable area meter (LICOR, Lincoln, NE, USA). The shoots and roots were separated for fresh weight (SFW and RFW, respectively) determinations and placed in an oven run at 105 °C for 30 min and then 80 °C until constant weight (for 48 h) to record shoots and roots dry weight (SDW and RDW, respectively). 

### 4.3. Assessment of Physiological and Biochemical Traits

#### 4.3.1. Chlorophyll Content (SPAD)

Chlorophyll content was determined with soil and plant analyzer development (SPAD) value on the third top fully expanded leaf using a chlorophyll meter (Minolta, Japan) which provides a rapid, accurate, and non-destructive estimate of leaf chlorophyll content. Nine data per replicate with three replicates in each treatment were collected [72]. 

#### 4.3.2. Determination of K^+^, Proline, and Total Soluble Sugars Concentrations

K^+^ was measured using a flame photometer [73]. Proline concentration in the dried specimen was assessed using a rapid colorimetric method as described by Bates et al. 1973 [74]. Total soluble sugars were assessed using the anthrone method [75]. 

#### 4.3.3. Determination of Water Relations and Electrolyte Leakage 

Using fresh fully-expanded leaves excluding the midrib, assessments of relative water content [76] and electrolyte leakage [77] were conducted.

#### 4.3.4. Assessment of Antioxidant Enzymes Activity

Fresh specimen of the second fully expanded youngest leaves were collected from the seedling and stored at –80 °C until analysis to determine the biochemical indices; catalase (CAT; EC 1.11.1.6), superoxide dismutase (SOD; EC 1.15.1.1) peroxidase (POD; EC 1.11.1.7), and ascorbate peroxidase (APX; EC 1.11.1.11). 0.5 g samples were homogenized in ice-bath with 4.5 ml of 100 mM potassium phosphate buffer (pH 7.0) containing 1% polyvinylpyrrolidone (PVP) (w/v) and 0.05% Triton X-100 (v/v). For the extraction of APX, the extraction media was supplemented with 2 mM ascorbic acid [78]. Homogenates were centrifuged at 15,000× *g* for 20 min at 4 °C. All biochemical indices of leaves were determined using commercial ELISA kits following the manufacturer’s instructions (MEIMIAN, Jiangsu, China).

### 4.4. Salt Tolerance Evaluation

The comprehensive salt tolerance evaluation was performed based on membership function value (MFV) according to the previous report [31]. MFV was calculated from the salt tolerance coefficients (or indexes). Salt tolerance coefficients (STC) were calculated using the formula [31]

STC = (ST/CK) × 100(1)

where CK is the mean value of a single trait under the control treatment and ST is the mean value of a single trait under salinity treatment. 

STC subjected to statistical data analysis for all the traits; morphological, physiological, and biochemical traits. The MFV of salt tolerance was calculated using the formula (MFV1 and MFV2), Formula MFV1 was used for the indices that are directly related to salinity tolerance, and MFV2 was used for the traits that are inversely related indices as explained:

MFV1 (X*i*) = (X*i* − X min)/(X max − X min) (2)

MFV2 (X*i*) = 1 − (X*i* − X min)/(X max − X min) (3)

where X is the *i* NaCl tolerance coefficient, max is the maximum NaCl tolerance coefficient value from all cultivars of the *i* index, and X min is the minimum NaCl tolerance coefficient from all cultivars of the *i* index.

For the aim of ranking the degree of salinity tolerance among rapeseed cultivars (from 1 to 10), the mean of MFV was calculated as average of the membership function values of all traits for each cultivar. Grouping of rapeseed cultivars for salt tolerance was done by subject data of the MFV to hieratical cluster (Euclidean distance) analysis, and salinity tolerance was divided into three levels: salt-tolerant, moderately salt-tolerant, and salt-sensitive groups. Rapeseed cultivars were further subjected to photosynthetic gas exchange and anatomical traits analysis to assess its role in salinity tolerance of rapeseed cultivars. 

### 4.5. Measurement of Photosynthetic Gas Exchange Parameters

Leaf net photosynthetic rate (Pn), transpiration rate (Tr), and stomatal conductance (Gs) were assessed for photosynthetic gas exchange parameters using a portable photosynthesis system (LI-6400, United States). The assessments were taken between 9:00 and 11:00 a.m. on the third top leaf. The CO_2_ concentration of the leaf chamber was 400 µmol mol^−1^. The airflow speed was 500µmol s^−1^. The photosynthetically active radiation (PAR) was 1000 µmol m^−2^ s^−1^. The leaf temperature was 24 ± 2° C, and the air relative humidity was 70–80%. The data were automatically collected every 2–3 min with at least five replicates.

### 4.6. Assessment of Stomata Anatomical Parameters 

To estimate the density and stomatal aperture area measurements, three plants from each replicate (nine plants of each treatment) were selected. Measurement and scoring were performed on third leaf from the apex of each plant. One sample of epidermal cells was obtained from the lower surface (abaxial side) by the nail varnish technique [79]. A small area of abaxial side of leaves was covered with a thin layer of clear nail polish and placed on glass slide and left to dry after drying the leaves samples removed and observed through a light microscope (BX60, Olympus, Hamburg, Germany), equipped with a digital camera (Camedia C4040, Olympus, Hamburg, Germany) [80]. Stomata density and stomatal aperture area were measured with the AnalySIS^®^ 3.2 software program for image analysis.

### 4.7. Analysis of Data

Data were analyzed using Genstat 17th edition software package [81]. Where significant differences were detected, means were separated using the least significant difference (LSD) test procedure at the 5% significance level using the Duncan’s Multiple Range Test. The quantified salinity tolerance ranking and cluster analysis were also performed using Genstat 17th edition. The graphical presentation was carried out using Origin 8, and R 3.5.1 was used to calculate the Pearson’s correlation coefficient.

## 5. Conclusions

The membership function value (MFV) based on different morpho-physiological and biochemical traits could be used as a new tool for screening rapeseed for salinity tolerance. MFV and Euclidean cluster analysis showed that Hua6919 and Yunyoushuang2 were the most salt-tolerant while Zhongshuang11 and Yangyou9 were the most salt-sensitive cultivars. Moreover, this study has provided evidence that photosynthetic gas exchange parameters and stomatal aperture are strongly correlated with salinity tolerance. Among the photosynthetic gas exchange and anatomical traits, the stomatal aperture was the most highly correlated with salinity tolerance in rapeseed cultivars and thus, is important for future studies that aim to improve salinity tolerance in rapeseed. The most salt-tolerant cultivars can be used as target cultivars for breeding programs that aim to improve salt tolerance in the future. 

## Figures and Tables

**Table 1:** Means of salt tolerance coefficients * based on growth and biomass parameters in *B. napes* cultivars.

Cultivars	PH	TLA	NL	SFW	RFW	SDW	RDW
Zhongshuang11	71.7	39.1	77.8	41.5	48.9	45.7	46.2
Yangyou9	69.2	45.6	70.5	47.2	57.1	48.2	44.3
Hua6919	82.9	57.4	88.2	52.7	66.8	74.3	65.7
Xiangyouza553	73.8	48.8	71.4	49.1	49.1	52.3	49.7
Yangza11	80.2	44.5	70.3	42.6	53.1	49.1	50.4
Fengyou520	88.5	60.6	77.1	54.2	62.6	72.3	61.7
Huashuang5	76.7	53.7	78.2	48.3	62.1	64.2	52.8
Ningza27	71.2	43.8	71.5	37.6	54.7	52.2	53.0
Rongyou18	77.5	49.5	86.2	47.4	57.6	60.1	53.7
Yunyoushuang2	83.5	56.9	89.1	53.2	65.2	65.5	57.6

* Means of salt tolerance coefficients were defended as the average of the observations under two salt stresses divided by the means of the controls and expressed as a percentage. PH; plant height, TLA; total leaves area, NL; number of leaves, SFW; shoot fresh weight, RFW; root fresh weight, SDW; shoot dry weight, RDW; root dry weight.

**Table 2:** Means of salt tolerance coefficients * based on physiological and biochemical traits in *B. napes* cultivars

Cultivars	SPAD	K^+^	TSS	ProC	RWC	EL	SOD	CAT	APX	POD
Zhongshuang11	100	16.2	134	147	76.5	190.6	149	159	103	135
Yangyou9	85	15.9	130	159	77.0	173.1	180	141	94	144
Hua6919	119	25.6	158	177	88.2	121.0	245	177	121	182
Xiangyouza553	96	15.8	134	172	81.0	184.8	165	137	105	128
Yangza11	104	17.5	134	169	80.2	139.1	176	143	94	133
Fengyou520	129	22.9	159	202	84.7	170.1	192	195	100	193
Huashuang5	115	23.9	145	197	84.6	189.2	130	138	116	179
Ningza27	111	19.9	126	179	84.3	187.5	164	144	120	136
Rongyou18	120	21.5	139	189	83.9	144.4	174	183	112	154
Yunyoushuang2	121	24.9	153	199	86.7	132.4	232	172	120	186

* Means of salt tolerance coefficients ware defended as the average of the observations under two salt stresses divided by the means of the controls and expressed as a percentage. SPAD; total chlorophyll, K^+^; potassium concentration, TSS; total soluble sugar, ProC; proline concentration, RWC relative water content, El; electrolyte leakage, SOD; superoxide dismutase, CAT; catalase, APX; ascorbate peroxidase, POD; peroxidase.

**Table 3:** Means of salt tolerance coefficient * of net photosynthetic rate (Pn), transpiration rate (Tr), and stomatal conductance (Gs), stomata aperture area (SAA), and stomatal density (SD) in *B. napes* cultivars.

Cultivars	Pn	Tr	Gs	SAA	SD
Zhongshuang11	43.6	43.6	32.2	26.0	129.2
Yangyou9	53.1	48.2	47.0	36.5	123.6
Hua6919	75.6	74.7	67.3	77.4	123.0
Xiangyouza553	45.0	42.9	33.8	48.8	124.8
Yangza11	44.5	49.4	38.3	40.8	122.8
Fengyou520	54.4	55.2	41.2	52.2	116.4
Huashuang5	63.8	66.8	52.4	55.8	112.1
Ningza27	50.6	51.6	45.9	46.7	135.8
Rongyou18	64.8	60.6	54.7	50.9	117.2
Yunyoushuang2	74.3	66.6	55.6	64.9	119.5

* Means of salt tolerance coefficients (indexes) ware defended as the average of the observations under two salt stresses divided by the means of the controls and expressed as a percentage.

## Supplementary Materials

The following are available online at https://www.mdpi.com/2223-7747/9/1/62/s1, Figure S1: Effect of different salt stress levels on chlorophyll content (SPAD). Figure S2: Effect of different salt stress levels on (A) K+ concentration, (B) total soluble sugar, and (C) proline concentration of *B. napus* L. cultivars. Figure S3: Effect of different salt stress levels on (A) relative water content (RWC%) and (B) electrolyte leakage (EL%) on *B. napus* L. cultivars. Figure S4. Effect of salt stress on antioxidants enzymes; (A) superoxide dismutase, (B) catalase, (C) ascorbate peroxidase, and (D) peroxidase of *B. napus* L. cultivars. Figure S5. Effect of salt stress on (A) net photosynthetic rate, (B) transpiration rate, and (C) stomatal conductance in *B. napus* L. cultivars. Figure S6. Effect of salt stress on (A) stomata aperture area and (B) stomata density in *B. napus* L. cultivars. Table S1. Effect of salt stress on plant height, number of leaves, and total leaves area of *B. napus* L. cultivars. Table S2. Effect of salt stress on the shoot and root fresh weights and shoot and root dry weights of *B. napus* L. cultivars. Table S3. Membership function value (MFV) of rapeseed cultivars.
