# Supplementary material for: Stomatal and Photosynthetic Traits Are Associated with Investigating Sodium Chloride Tolerance of Brassica napus L. Cultivars
Source: Plants (Basel). 2020 Jan 2;9(1):62. doi: 10.3390/plants9010062 (PMC7020420; doi:10.3390/plants9010062)
Supplement: Supplementary file 1 [file plants-09-00062-s001.zip › supplementary files/Supplementary figures.docx]

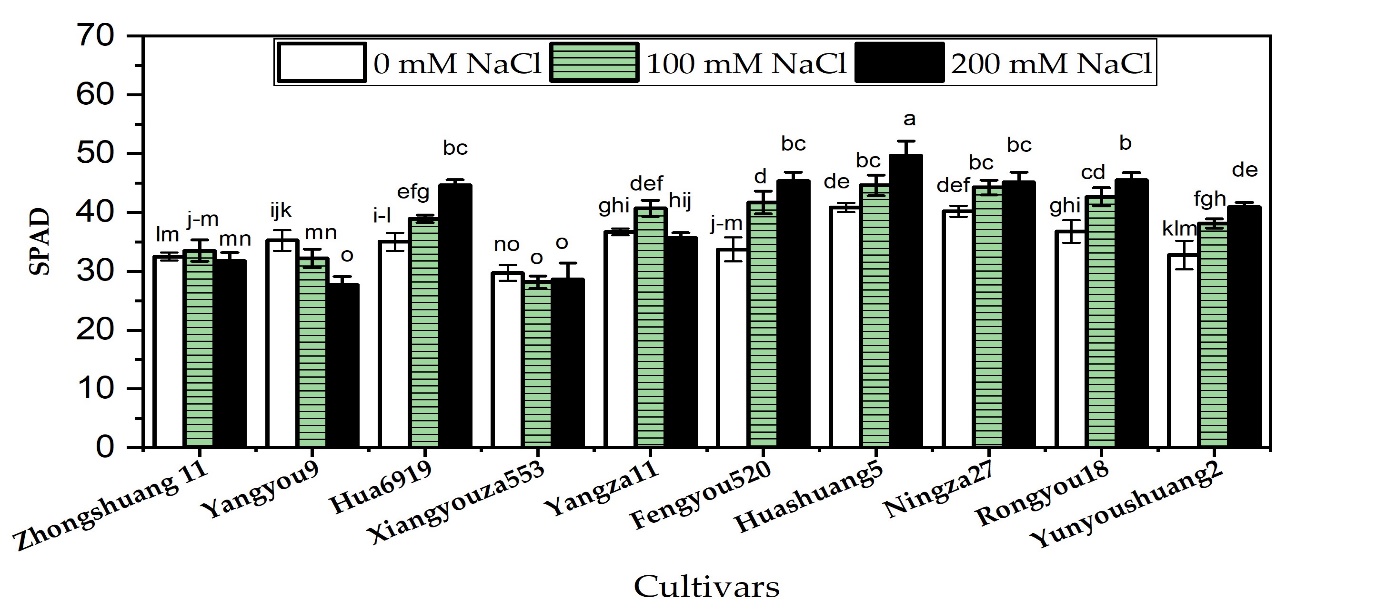


**Figure S1.** Effect of different salt stress levels on chlorophyll content (SPDA) of *B. napus* L. cultivars., Mean (±SD) was calculated from three replicates for each treatment. Bars with different letters are significantly at *P<*0.05 applying Duncan's Multiple Range Test.


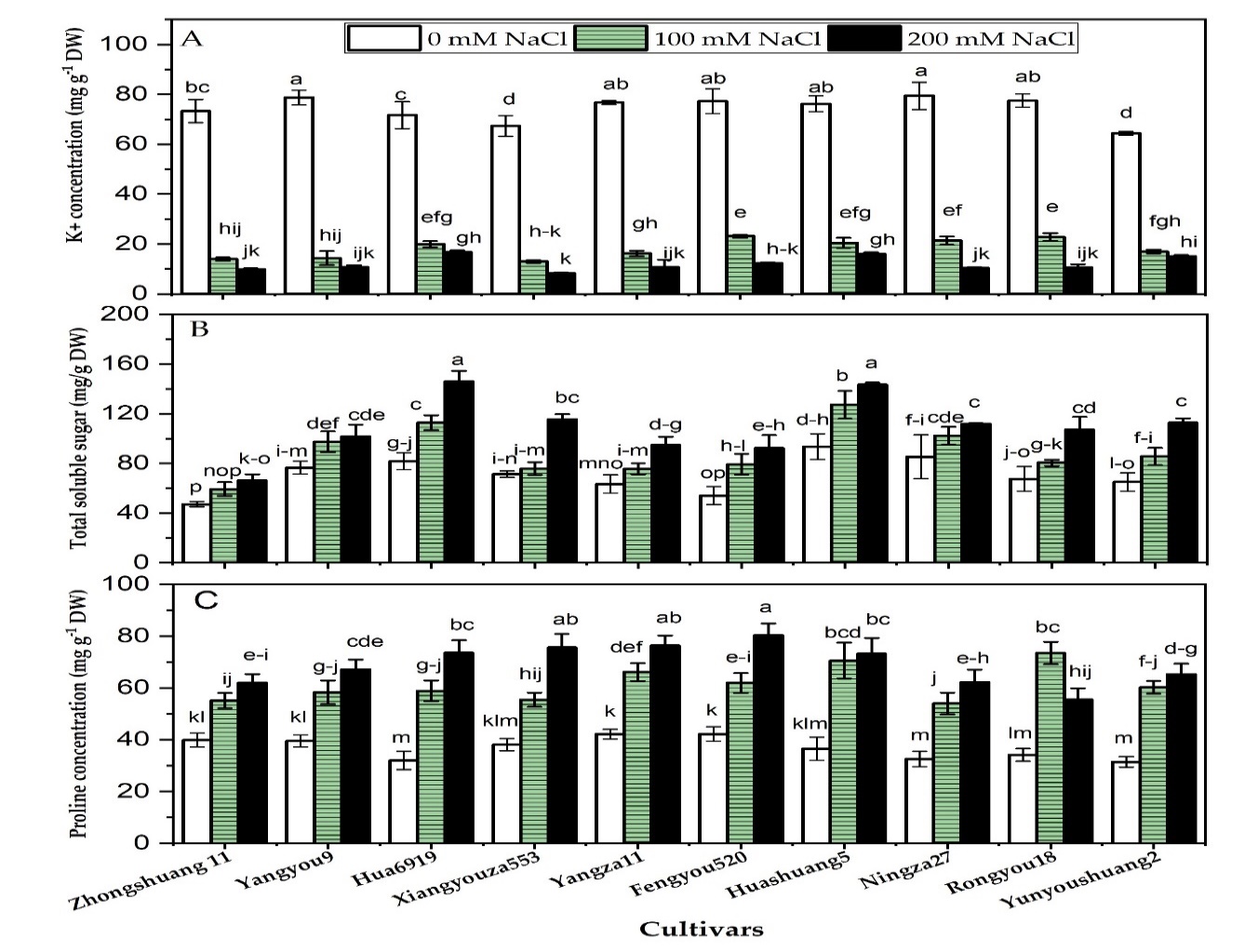


**Figure S2.** Effect of different salt stress levels on (A) K^+^ concentration, (B) total soluble sugar, and (C) proline concentration of *B. napus* L. cultivars. Mean (±SD) was calculated from three replicates for each treatment. Bars with different letters are significantly at *P<*0.05 applying Duncan's Multiple Range Test.


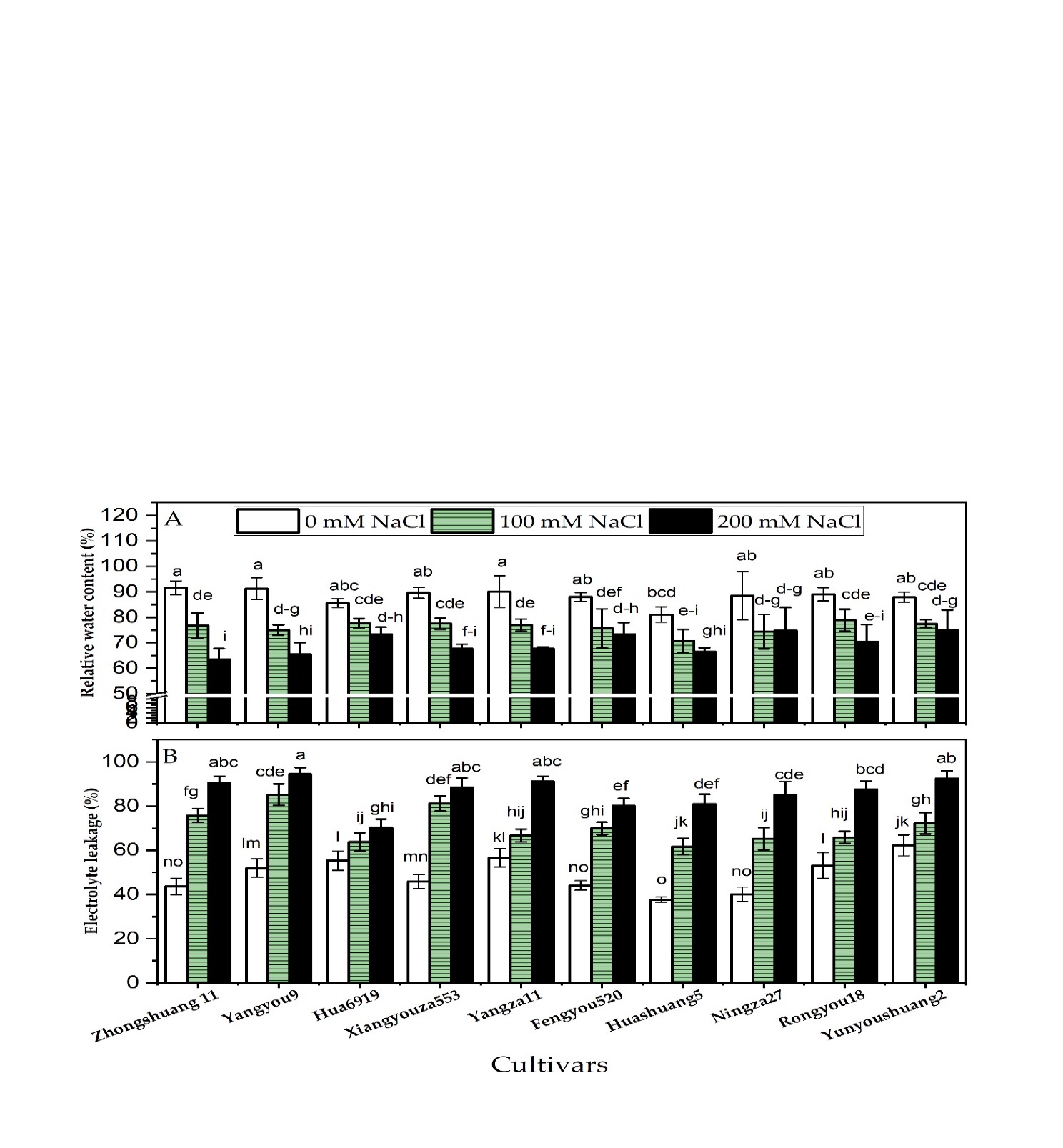


**Figure S3.** Effect of different salt stress levels on (A) relative water content (RWC%) and (B) electrolyte leakage (EL%) on *B. napus* L. cultivars. Mean (±SD) was calculated from three replicates for each treatment. Bars with different letters are significantly at *P<*0.05 applying Duncan's Multiple Range Test.


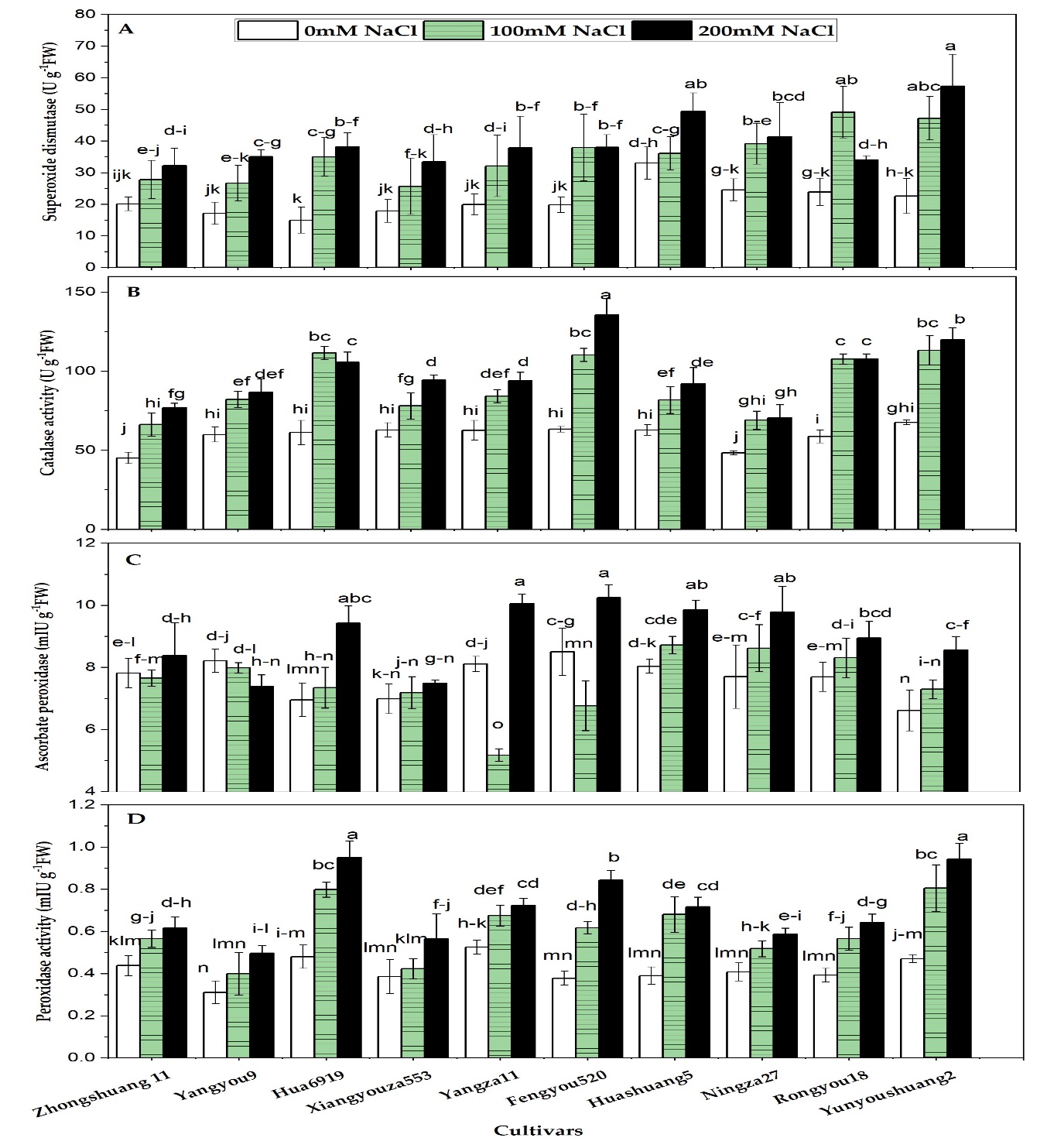


**Figure S4.** Effect of salt stress on antioxidants enzymes; (A) superoxide dismutase, (B) catalase, (C) ascorbate peroxidase, and (D) peroxidase of *B. napus* L. cultivars. Mean (±SD) was calculated from three replicates for each treatment. Bars with different letters are significantly at *P<*0.05 applying Duncan's Multiple Range Test.


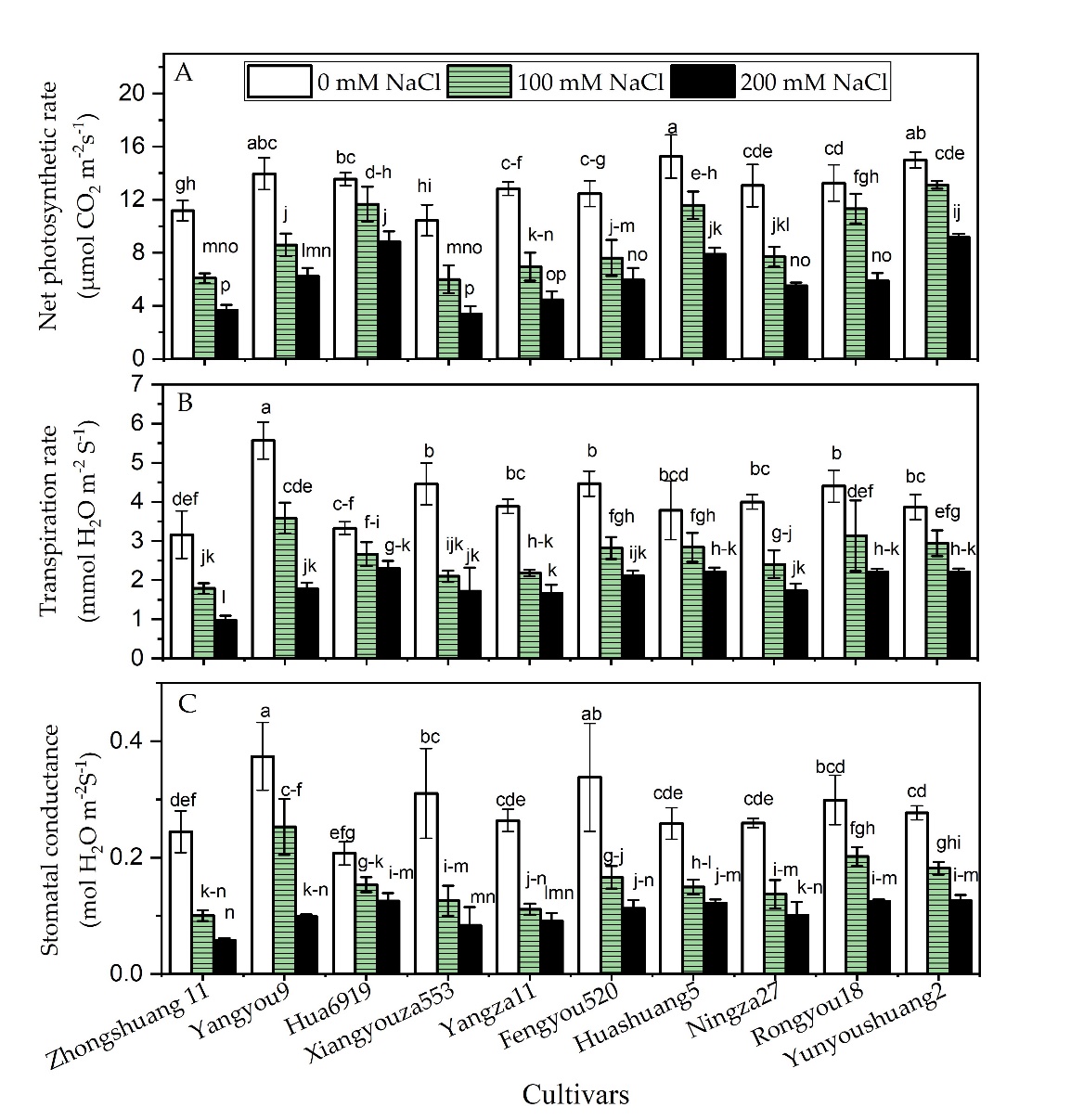


**Figure S5.** Effect of salt stress on (A) net photosynthetic rate, (B) transpiration rate, and (C) stomatal conductance in *B. napus* L. cultivars. Mean (±SD) was calculated from three replicates for each treatment. Bars with different letters are significantly at *P<*0.05 applying Duncan's Multiple Range Test.


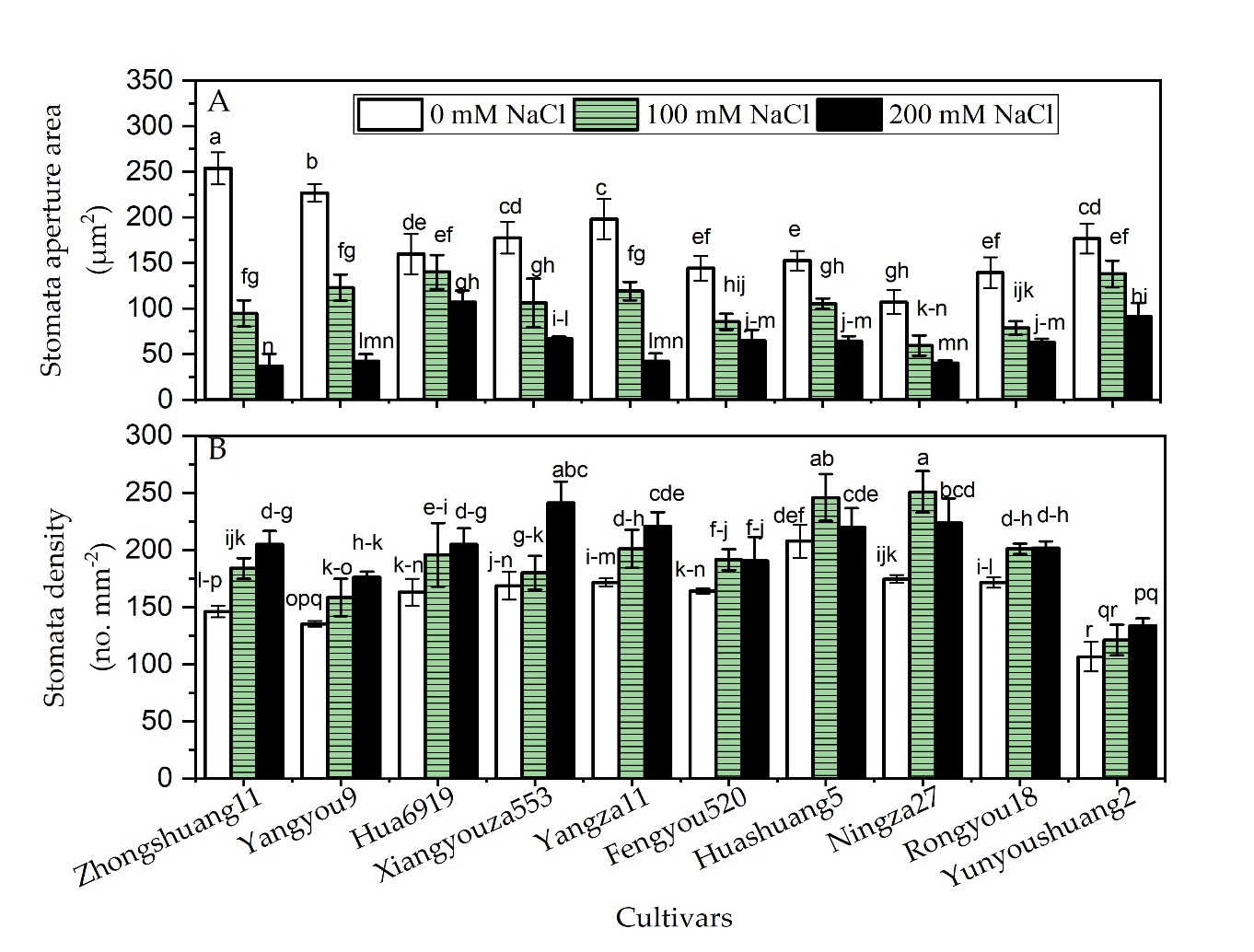


**Figure S6.** Effect of salt stress on (A) stomata aperture area and (B) stomata density in *B. napus* L. cultivars. Mean (±SD) was calculated from three replicates for each treatment. Bars with different letters are significantly at *P<*0.05 applying Duncan's Multiple Range Test.
