# Supplementary material for: Stomatal and Photosynthetic Traits Are Associated with Investigating Sodium Chloride Tolerance of Brassica napus L. Cultivars
Source: Plants (Basel). 2020 Jan 2;9(1):62. doi: 10.3390/plants9010062 (PMC7020420; doi:10.3390/plants9010062)
Supplement: Supplementary file 1 [file plants-09-00062-s001.zip › supplementary files/Supplementary tables.docx]

**Table S1.** Effect of salt stress on plant height, number of leaves, and total leaves area of *B. napus* L. cultivars.

| Cultivars (Cv.) | Plant height (cm) | | | Number of leaves (no. plant^-1^) | | | Total leaves area (cm^2^ plant^-1^) | | |
| --- | --- | --- | --- | --- | --- | --- | --- | --- | --- |
|  | Salinity level (S) | | | | | | | | |
|  | S0 | S1 | S2 | S0 | S1 | S2 | S0 | S1 | S2 |
| Zhongshuang11 | 55.1±1.7^bcd^ | 42.4±2.3^jkl^  (23) | 36.6±1.4^n^  (34) | 6.00±0.0^cde^ | 5.06±0.4^fgh^  (16) | 4.28±0.3^hi^  (29) | 164±8^d^ | 76±3^i-l^  (53) | 52±8^l^  (69) |
| Yangyou9 | 54.6±3.8^bcd^ | 42.3±0.8^jkl^  (22) | 33.2±3.0^o^  (39) | 6.11±0.8^cde^ | 4.83±0.6^gh^  (21) | 3.78±0.2^ij^  (38) | 165±29^d^ | 87±2^h-k^  (47) | 63±8^jkl^  (62) |
| Hua6919 | 50.6±1.4^ef^ | 44.7±0.7^hij^  (12) | 39.2±1.1^lmn^  (23) | 6.61±0.5^bc^ | 6.06±0.5^cde^  (8) | 5.61±0.4^d-g^  (15) | 165±13^d^ | 117±17^e-h^  (29) | 72±23^jkl^  (56) |
| Xiangyouza553 | 54.5±3.3^bcd^ | 43.0±0.8^ijk^  (21) | 37.4±2.7^mn^  (31) | 6.50±0.9^bc^ | 4.83±0.7^gh^  (26) | 4.44±0.1^hi^  (32) | 130±16^efg^ | 65±3^jkl^  (50) | 62±28^jkl^  (52) |
| Yangza11 | 54.1±2.3^cd^ | 47.1±2.3^fgh^  (13) | 39.6±0.3^k-n^  (27) | 5.61±0.7^d-g^ | 4.39±0.6^hi^  (22) | 3.50±0.4^j^  (38) | 163±4^d^ | 86±35^h-l^  (47) | 59±17^kl^  (64) |
| Fengyou520 | 48.9±1.3^efg^ | 46.5±0.6^gh^  (5) | 40.1±1.3^k-n^  (18) | 7.28±0.7^ab^ | 5.83±0.2^c-f^  (20) | 5.39±0.3^efg^  (26) | 140±10^def^ | 97±18^g-j^  (31) | 73±9^jkl^  (48) |
| Huashuang5 | 54.9±2.0^bcd^ | 46.1±2.0^ghi^  (16) | 38.2±2.0^mn^  (31) | 7.78±0.5^a^ | 6.33±0.2^cd^  (19) | 5.83±0.2^c-f^  (25) | 217±41^b^ | 144±9^de^  (34) | 90±18^h-k^  (59) |
| Ningza27 | 60.1±2.4^a^ | 48.1±1.1^fgh^  (20) | 37.6±1.5^mn^  (38) | 7.61±0.5^a^ | 5.78±0.4^c-f^  (24) | 5.11±0.3^fgh^  (33) | 276±16^a^ | 145±21^de^  (47) | 97±10^g-j^  (65) |
| Rongyou18 | 58.1±3.2^ab^ | 49.3±1.4^efg^  (15) | 40.7±1.0^klm^  (30) | 6.06±0.3^cde^ | 5.56±0.3^d-g^  (8) | 4.89±0.3^gh^  (19) | 202±10^bc^ | 118±9^e-h^  (41) | 81±21^i-l^  (60) |
| Yunyoushuang2 | 56.7±1.9^bc^ | 51.8±2.2^de^  (9) | 42.8±1.2^i-l^  (24) | 6.61±0.4^bc^ | 6.17±0.2^cde^  (7) | 5.61±0.2^d-g^  (15) | 247±26^a^ | 174±30^cd^  (30) | 108±3^f-i^  (56) |
| ANOVA |  | | |  | | |  | | |
| Cv. | *** | | | *** | | | *** | | |
| S | *** | | | *** | | | *** | | |
| Cv. x S | ** | | | ns | | | *** | | |

Values are means ± standard division (SD). Values with different letters are significantly different at *P*<0.05 (n=3). Values given in parenthesis indicate the percent (%) reduction as compared to control. S0, S1, and S2 indicate salinity levels of 0, 100, and 200 mM NaCl, respectively. *; significance at *p* ≤ 0.05; **; significance at *p* ≤ 0.01, ***; significance at *p* ≤ 0.001 and ns; no significance.

**Table S2.** Effect of salt stress on the shoot and root fresh weights and shoot and root dry weights of *B. napus* L. cultivars.

| Cultivars  (Cv.) | Shoot fresh weight (g plant^-1^) | | | Root fresh weight (g plant^-1^) | | | Shoot dry weight (g plant^-1^) | | | Root dry weight (g plant^-1^) | | |
| --- | --- | --- | --- | --- | --- | --- | --- | --- | --- | --- | --- | --- |
|  | Salinity levels (S) | | | | | | | | | | | |
|  | S0 | S1 | S2 | S0 | S1 | S2 | S0 | S1 | S2 | S0 | S1 | S2 |
| Zhongshuang11 | 6.06±0.3^f^ | 2.84±0.2^j^  (53) | 2.18±0.1^j^  (64) | 0.76±0.03^c^ | 0.43±0.02^hi^  (43) | 0.31±0.02^kl^  (59) | 0.422±0.01^ef^ | 0.224±0.02^m^  (47) | 0.161±0.01^n^  (62) | 0.062±0.008^de^ | 0.033±0.002^klm^  (47) | 0.024±0.002^n^  (61) |
| Yangyou9 | 7.26±0.3^e^ | 3.96±0.2^hi^  (46) | 2.89±0.2^j^  (60) | 0.64±0.03^def^ | 0.42±0.03^hi^  (35) | 0.32±0.03^kl^  (51) | 0.518±0.02^d^ | 0.286±0.02^kl^  (45) | 0.214±0.01^mn^  (59) | 0.080±0.005^c^ | 0.042±0.004^hij^  (47) | 0.029±0.004^lmn^  (64) |
| Hua6919 | 8.20±0.4^d^ | 4.92±0.5^g^  (40) | 3.74±0.5^i^  (54) | 0.86±0.07^b^ | 0.68±0.03^d^  (21) | 0.47±0.03^gh^  (45) | 0.539±0.01^d^ | 0.446±0.03^e^  (17) | 0.354±0.04^hij^  (34) | 0.082±0.008^c^ | 0.065±0.007^d^  (21) | 0.043±0.003^hi^  (48) |
| Xiangyouza553 | 5.07±0.3^g^ | 2.88±0.4^j^  (43) | 2.10±0.2^j^  (59) | 0.77±0.04^c^ | 0.40±0.02^ij^  (48) | 0.35±0.01^jk^  (54) | 0.384±0.02^e-i^ | 0.243±0.03^lm^  (37) | 0.159±0.02^n^  (59) | 0.068±0.005^d^ | 0.037±0.002^ijk^  (45) | 0.031±0.002^k-n^  (55) |
| Yangza11 | 7.71±0.3^de^ | 3.74±0.3^i^  (52) | 2.83±0.2^j^  (63) | 0.60±0.02^f^ | 0.36±0.02^jk^  (40) | 0.28±0.02^l^  (54) | 0.531±0.02^d^ | 0.311±0.01^jk^  (41) | 0.210±0.02^mn^  (60) | 0.061±0.003^def^ | 0.035±0.005^jkl^  (42) | 0.026±0.003^mn^  (57) |
| Fengyou520 | 9.20±0.7^c^ | 5.87±0.5^f^  (36) | 4.11±0.4^hi^  (55) | 0.62±0.04^ef^ | 0.46±0.02^hi^  (26) | 0.32±0.02^kl^  (48) | 0.418±0.00^efg^ | 0.345±0.02^ij^  (17) | 0.259±0.02^klm^  (38) | 0.064±0.005^d^ | 0.047±0.005^gh^  (27) | 0.033±0.004^klm^  (49) |
| Huashuang5 | 10.5±0.4^b^ | 5.97±0.2^f^  (43) | 4.13±0.2^hi^  (61) | 0.86±0.04^b^ | 0.64±0.02^def^  (25) | 0.42±0.03^hi^  (51) | 0.753±0.08^b^ | 0.553±0.04^d^  (27) | 0.414±0.04^e-h^  (45) | 0.098±0.003^ab^ | 0.056±0.001^ef^  (43) | 0.048±0.004^gh^  (51) |
| Ningza27 | 13.5±0.8^a^ | 5.85±0.2^f^  (57) | 4.32±0.3^ghi^  (68) | 0.93±0.08^a^ | 0.62±0.04^ef^  (34) | 0.40±0.01^ij^  (57) | 0.896±0.07^a^ | 0.563±0.07^d^  (37) | 0.372±0.03^f-j^  (58) | 0.105±0.004^a^ | 0.064±0.003^d^  (39) | 0.047±0.003^gh^  (55) |
| Rongyou18 | 9.09±0.9^c^ | 4.71±0.1^gh^  (48) | 3.91±0.2^hi^  (57) | 0.83±0.02^b^ | 0.52±0.04^g^  (37) | 0.43±0.04^hi^  (48) | 0.619±0.04^c^ | 0.387±0.02^e-i^  (37) | 0.358±0.03^g-j^  (42) | 0.093±0.005^b^ | 0.056±0.003^ef^  (40) | 0.044±0.003^hi^  (53) |
| Yunyoushuang2 | 10.8±1.0^b^ | 7.03±0.8^e^  (35) | 4.50±0.2^ghi^  (58) | 0.86±0.04^b^ | 0.66±0.02^de^  (23) | 0.46±0.02^hi^  (46) | 0.804±0.01^b^ | 0.632±0.02^c^  (21) | 0.421±0.04^ef^  (48) | 0.105±0.007^a^ | 0.068±0.003^d^  (36) | 0.053±0.002^fg^  (49) |
| ANOVA |  | | |  | | |  | | |  | | |
| Cv. | *** | | | *** | | | *** | | | *** | | |
| S | *** | | | *** | | | *** | | | *** | | |
| Cv. x S | *** | | | *** | | | *** | | | *** | | |

Values are means ± standard division (SD). Values with different letters are significantly different at *P*<0.05 (n=3). Values given in parenthesis indicate the percent (%) reduction as compared to control. S0, S1, and S2 indicate salinity levels of 0, 100, and 200 mM NaCl, respectively. *; significance at *p* ≤ 0.05; **; significance at *p* ≤ 0.01, ***; significance at *p* ≤ 0.001 and ns; no significance.

**Table S3.** Membership function value (MFV) of rapeseed cultivars.

| **Cultivars** | **PH** | **TLA** | **NL** | **SFW** | **RFW** | **SDW** | **RDW** | **SPAD** | **K^+^** | **TSS** | **ProC** | **RWC** | **EL** | **SOD** | **CAT** | **APX** | **POD** | **Mean** | **Ranking** |
| --- | --- | --- | --- | --- | --- | --- | --- | --- | --- | --- | --- | --- | --- | --- | --- | --- | --- | --- | --- |
| Zhongshuang11 | 0.13 | 0.00 | 0.40 | 0.23 | 0.00 | 0.00 | 0.09 | 0.35 | 0.04 | 0.24 | 0.00 | 0.00 | 0.00 | 0.17 | 0.38 | 0.33 | 0.10 | 0.14 | 10 |
| Yangyou9 | 0.00 | 0.30 | 0.00 | 0.58 | 0.46 | 0.09 | 0.00 | 0.00 | 0.01 | 0.13 | 0.21 | 0.04 | 0.25 | 0.44 | 0.07 | 0.00 | 0.25 | 0.17 | 9 |
| Hua6919 | 0.71 | 0.85 | 0.96 | 0.91 | 1.00 | 1.00 | 1.00 | 0.78 | 1.00 | 0.98 | 0.54 | 1.00 | 1.00 | 1.00 | 0.70 | 1.00 | 0.83 | 0.90 | 1 |
| Xiangyouza553 | 0.24 | 0.45 | 0.06 | 0.69 | 0.01 | 0.23 | 0.25 | 0.24 | 0.00 | 0.26 | 0.45 | 0.38 | 0.08 | 0.31 | 0.00 | 0.42 | 0.00 | 0.24 | 8 |
| Yangza11 | 0.57 | 0.25 | 0.00 | 0.30 | 0.23 | 0.12 | 0.28 | 0.43 | 0.17 | 0.27 | 0.40 | 0.32 | 0.74 | 0.4 | 0.10 | 0.01 | 0.07 | 0.27 | 7 |
| Fengyou520 | 1.00 | 1.00 | 0.37 | 1.00 | 0.76 | 0.93 | 0.81 | 1.00 | 0.73 | 1.00 | 1.00 | 0.70 | 0.29 | 0.54 | 1.00 | 0.24 | 1.00 | 0.79 | 3 |
| Huashuang5 | 0.39 | 0.68 | 0.43 | 0.64 | 0.74 | 0.65 | 0.40 | 0.69 | 0.83 | 0.58 | 0.90 | 0.69 | 0.02 | 0.00 | 0.02 | 0.82 | 0.78 | 0.55 | 5 |
| Ningza27 | 0.10 | 0.22 | 0.07 | 0.00 | 0.32 | 0.23 | 0.41 | 0.6 | 0.42 | 0.00 | 0.57 | 0.67 | 0.04 | 0.30 | 0.13 | 0.96 | 0.12 | 0.30 | 6 |
| Rongyou18 | 0.43 | 0.48 | 0.86 | 0.59 | 0.48 | 0.5 | 0.44 | 0.79 | 0.58 | 0.40 | 0.76 | 0.63 | 0.66 | 0.38 | 0.81 | 0.69 | 0.40 | 0.58 | 4 |
| Yunyoushuang2 | 0.74 | 0.83 | 1.00 | 0.94 | 0.91 | 0.69 | 0.62 | 0.81 | 0.93 | 0.82 | 0.95 | 0.87 | 0.84 | 0.88 | 0.61 | 0.97 | 0.89 | 0.84 | 2 |

PH; Plant height, TLA; Total leaves area, NL; Number of leaves, SFW; Shoot fresh weight, RFW; Root fresh weight, SDW; Shoot dry weight, RDW; Root dry weight, K^+;^ Potassium concentration, ProC; Proline concentration, TSS; Total soluble sugar, RWC; Relative water content, EL; Electrolyte leakage, SOD; Superoxide dismutase, APX; ascorbate peroxidase, CAT; catalase, POD; peroxidase.
